# Community outreach for patients who have difficulties in maintaining contact with mental health services: longitudinal retrospective study of the Japanese outreach model project

**DOI:** 10.1186/s12888-014-0311-y

**Published:** 2014-11-18

**Authors:** Mami Kayama, Yoshifumi Kido, Nozomi Setoya, Aki Tsunoda, Asami Matsunaga, Takahiro Kikkawa, Takashi Fukuda, Masayuki Noguchi, Keiko Mishina, Masaaki Nishio, Junichiro Ito

**Affiliations:** Psychiatric and Mental Health Nursing, College of Nursing, St. Luke’s International University, 10-1 Akashi-cho, Chuo-ku, Tokyo 104-0044 Japan; Department of Psychiatric Nursing, The University of Tokyo, Tokyo, Japan; Department of Nursing, School of Health Sciences, Tokai University, Kanagawa, Japan; National Institute of Public Health, Saitama, Japan; Okayama Psychiatric Medical Center, Okayama, Japan; Hanazono University, Clinical Psychology, Faculty of Social Welfare, Kyoto, Japan; Tohoku Fukushi University, Social Welfare, Faculty of General Welfare, Miyagi, Japan; National Center of Neurology and Psychiatry, National Institute of Mental Health, Psychiatric Rehabilitation, Tokyo, Japan

**Keywords:** Community psychiatry, Outcomes research, Readmission rate, Care length

## Abstract

**Background:**

Japan still has the highest ratio of beds devoted to psychiatric patients in the world. In 2011, in order to reduce re-hospitalization of patients who became disconnected from regular contact with outpatient medical services, the Japanese Ministry established the Japanese Outreach Model Project (JOMP). In this study, we will explicate the JOMP project protocol and investigate the rate and length of hospital admission, impairments of social function and problematic behavior at the follow-up period (6- and 12-month) and time of services provided by JOMP.

**Method:**

This longitudinal retrospective study used survey data collected from 32 outreach teams of 21 prefectures in Japan during September 2011 to July 2013. The outcome variables were assessed at baseline, 6-month and 12-month as to whether or not participants had been admitted to the hospital. Data from 162 participants with mental illness who had difficulties in maintaining contact with mental health services were analyzed. Repeated measures analysis of variance provided a significant effect of the intervention over time.

**Results:**

The rate of hospital admission of JOMP participants was 24.1% at 6-months and 27.2% at the 12-month follow-up. The average length of hospital-stay at baseline and 12-months was 38.7 days (SD 84.7). Compared with the baseline, the average score of the Global Assessment Functioning and the Social Behavioral Schedule were significantly improved after the 6-month and 12-month follow-up. The activity log showed that among the most often delivered JOMP services were to “prevent exacerbation of somatic symptoms” and “care for families”.

**Conclusion:**

These results suggest that JOMP has a strong potential to both reduce readmission rates and the length of hospital stay compared with the Japanese regular outpatient care by public insurance, and improve social function and problematic behavior. The JOMP teams provided long-term support for families. As of April 2014 JOMP was included in the National Health Insurance program in a limited way therefore an evaluation of JOMP team fidelity on readmissions must be examined.

## Background

Japan still has the highest ratio of beds (2.7 beds per 1,000 persons) devoted to psychiatric patients, in the world [1]. For decades hospitalization with custodial care was the only hospital care option particularly for people diagnosed with schizophrenia and other disabling mental disabilities [2]. By the late 1990’s and early 21^st^ century Japan was implementing mental health reform including deinstitutionalization and community mental health services [2]. While expanding community care for people with mental illness, remains a formal policy in most countries the extent to which community care is offered and the programming should be tied to the countries resources [3]. For a high resourced country such as Japan it is suggested that hospitalization be well balanced with community care and that active outreach to patients in the community is an important component of the mental health model. Models and outcomes of several community-based outreach programs have been implemented and widely researched such as assertive community treatment (ACT) [4-7] and assertive outreach (AO) [6,8-12].

Researchers have reported the outcomes of ACT implemented at various sites in Japan, ACT reduces hospital days [13], decreases the dosage of antipsychotics [14], and increases social functioning, self-efficacy and service satisfactions [15]. However Japan has no nation-wide implementation program for ACT. Furthermore there are many patients who have difficulties maintaining regular contact with medical services. They are neither integrated into the regular outpatient care by public insurance [16], nor have they become the target population of ACT. They are at a high risk for involuntary hospitalization. At the beginning of the 21^st^ century the average length of stay in psychiatric hospital in Japan is as long as 291.9 days [1,17], once admitted their length of stay would be prolonged.

In 2011, the Japanese Ministry of Health, Labor and Welfare established the Japanese Outreach Model Project (JOMP), which provides multidisciplinary outreach services for eligible patients to prevent them from repeated hospitalizations. Patients, who do not or will not use the services under the regular Japanese outpatient care funded by public insurance, yet are at high-risk for hospitalizations are the target population of JOMP. Multi-professional outreach teams implemented JOMP providing medical and social services including support for: daily living tasks, communications, mental and physical health, social life and family care. Services are provided 24 hours a day seven days a week (24/7) in the community setting.

Table 1 displays model elements of the JOMP compared to ACT, AO and the regular Japanese outpatient care. The target population of ACT and AO are restricted to such patients who are suffering from SMI or high users of mental health services and patients with difficulties maintaining contact with services; JOMP target populations are patients with difficulties maintaining contact with medical services. All services are multi-professional and ACT and JOMP include peer staff. All the services provide 24/7 services and use case management. Ratios of patient and clinical staff are as follows: 12:1 at ACT, more than 10:1–12:1 at AO and JOMP had a range from 3:1 to 20:1 with an average 6:1. Only the JOMP duration of patient contact must be several months or longer because service users are evaluated about the necessity of JOMP every 6 months. In Japan the regular outpatient care is provided at hospitals or clinics. If patients stopped their regular visits to psychiatrists, they easily tend to drop out of medical care. Patients can receive case management and home visits as an optional service of the regular outpatient care. By law the outpatient caseload of a psychiatrist is 80:1.

**Table 1:** **Comparison of model elements**

**Team/model characteristics**	**ACT (DACT)** ^**1)**^	**AO** ^**2)**^	**JOMP**	**Japanese regular outpatient care (Public insurance)** ^**3)**^
Target population	Patients suffering severe mental illness (SMI)	High users of mental health services	Quit their psychiatric outpatient treatment for more than 3 month	Patients with mental illness
Involved service providers	Multiprofessional	Multiprofessional psychiatrist, nurse and others	Psychiatrist, Nurse, PSW, OT, CP, Peer-staff’	Psychiatrist others: as necessary
24/7 service	Yes	Yes	Yes	No
Case management	Yes	Yes	Yes	Optional
Home treatment	Yes	Yes	Yes	No
Patient to clinical staff ratios	12:1	>10:1	3:1–20:1	80:1^4)^
Duration of relation	Long term	Long term	Evaluate every six month	Depends on patients

ACT, assertive community treatment; DACTS; dartmouth assertive community treatment scale.

AO, assertive outreach; JOMP, Japanese outreach model project.

SMI, severe mental illness; OT, occupational therapist; SW, social worker; CP, clinical psychologist.

1) Teague, Bond & Drake (1998). Program fidelity in assertive community treatment. American journal of orthopsychiatry, 68(2), 216–233.

2) Department of Health. (2001). Mental health policy implementation guide.

3) http://www.mhlw.go.jp/bunya/iryouhoken/iryouhoken15/dl/2-11.pdf (in Japanese).

4) http://wwwhourei.mhlw.go.jp/cgi-bin/t_docframe.cgi?MODE=tsuchi&DMODE=CONTENTS&SMODE=NORMAL&KEYWORD=&EFSNO=1154 (in Japanese).

The goal of this model project was to prevent hospitalization of persons with mental illness who fail to keep contact with medical services and to transfer them into the regular Japanese outpatient care financed by the public insurance system. This model project was a governmental trial and was initiated throughout Japan to enrich the community care.

The aim of this study was to evaluate the JOMP program and determine its impact on selected variables of the target population: 1) participants characteristics, 2) rate and length of hospital admission at the follow-up periods (6 and 12 months), 3) impairments of social function and symptoms at the follow-up periods (6 and 12 months) and 4) total amount of time of services provided by the JOMP team.

## Methods

### Design and sample

**Figure 1:**
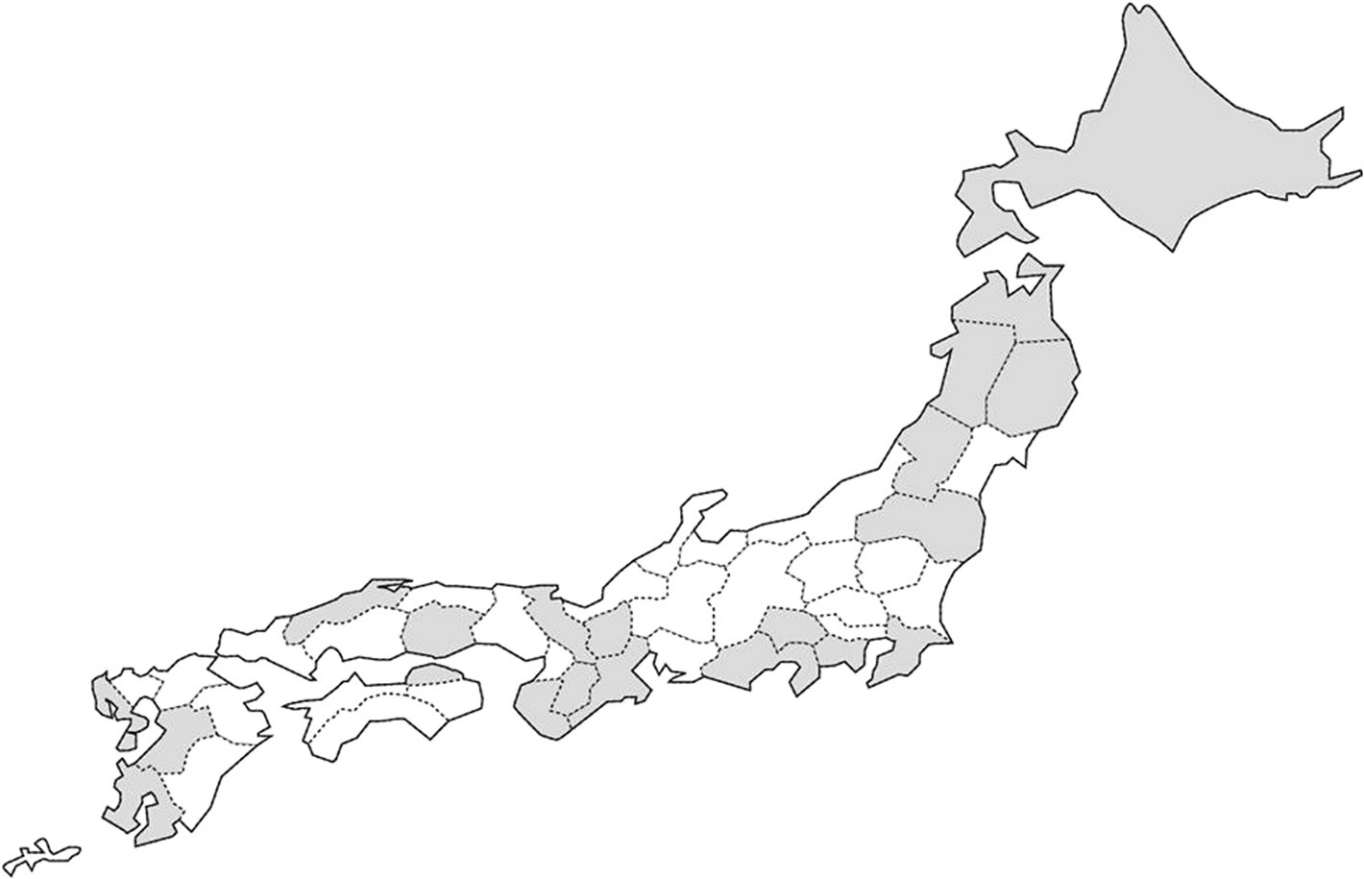
**The location of 32 participating teams in the 21 prefectures.**

The JOMP survey was a longitudinal epidemiological survey using a purposive sample. Of the 47 prefectures in Japan, 24 were implementing JOMP and 21 participated in the survey. Data were collected from 32 multi-professional outreach teams functioning in the 21 prefectures, agreeing to participate (see Figure 1).

Participants for JOMP were identified through the committee meeting consisting of the JOMP team members, local welfare commissioners, civil service workers, public health nurses, experts such as those in academic positions, local associations for mental health services and their families. Every six months, the committee evaluated patients as to whether they should continue to use JOMP service or be transfer to regular Japanese outpatient care.

There were 215 patients who met the inclusion criteria of dropping out of outpatient care for more than 3 months. However 53 were excluded because their service duration had not reached the first 6-month point in time. Therefore we analyzed 162 (75.3%) participants who completed baseline, and the two 6-month follow-up evaluations and daily activity logs. The study commenced September 2011 and continued for 22 months ending in July 2013.

### Setting

Each JOMP team was established as a unit of the department of outpatient care of a psychiatric hospital, visiting nurse station, and community activity support centers. The team consisted of a psychiatrist, nurse, social worker, occupational therapist, psychologist, peer staff, and medical clerk. The catchment area was defined as ‘within a 30- minute driving time’. The JOMP multidisciplinary care teams provided the following services: 1) creation of a care plan and case management, 2) support for daily living and acquisition of life skills, 3)support for building and dealing with interpersonal relationships, 4) support for families, 5) support for management of psychiatric symptoms, 6) support for managing somatic symptoms, 7) support for social living, 8) support for the living environment, 9) support for work and education, and 10) empowerment via outreach services The project included consultation, case conferences, first access, assessment, planning, and care delivery (include crisis solution). Those services had been delivered for patients’ at their homes and patients were encouraged to seek the outpatient care facilities first or as early as possible. If patients would continue their relationships with the outpatient care facilities, then JOMP team encouraged them to go to outpatient department with a member of JOMP team There was no particular programmed home treatment but JOMP delivered basic case management and care for building trust with patients who dropped out from regular Japanese outpatient clinic funded by public insurance.

### Ethical considerations

The JOMP teams were informed of the purpose, methods, measurements and right to withdraw from the study without penalty. All data were collected anonymously by using participant’s IDs and staff member IDs. They understood their anonymity would be protected when presenting or publishing the results. The Research Ethics Committee of St. Luke’s College of Nursing approved this study (11–032).

### Procedures and measures

**Figure 2:**
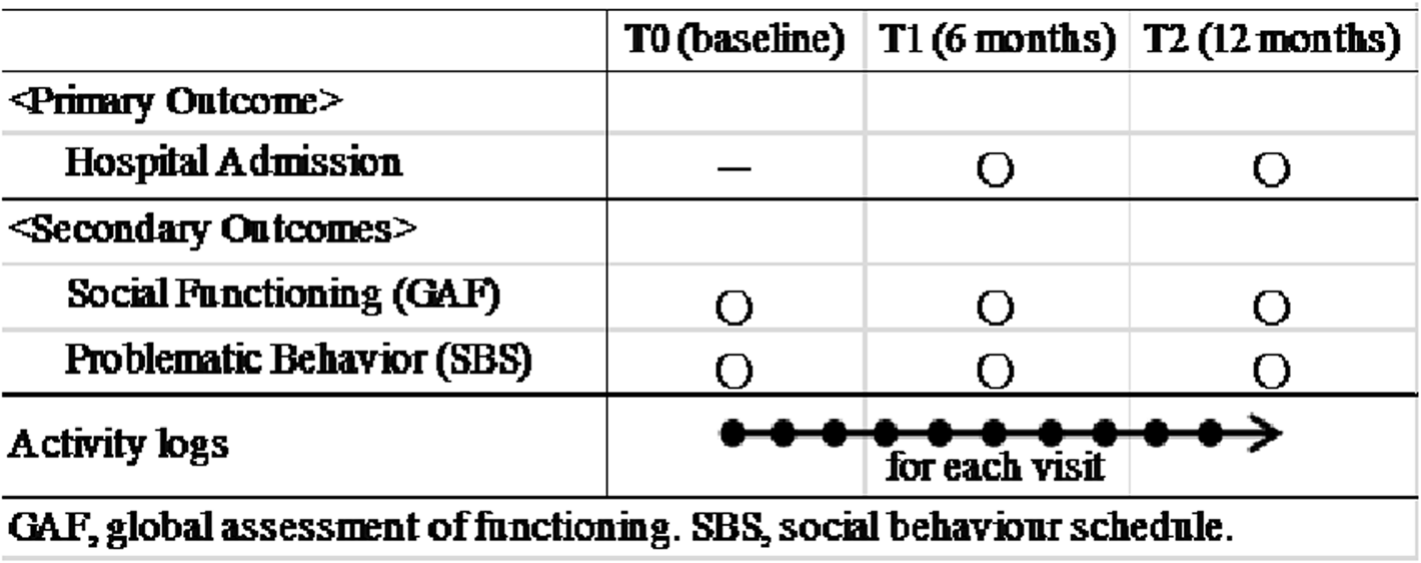
**Timeline of measurement.**

Data were collected at three points in time. At the baseline (T0), the characteristics of the participants were assessed, including diagnosis, sociodemographic data (age, gender, marital status, living situation and occupation), hospitalization and medication during the past 18-months, social functioning and problematic behavior. The outcomes were assessed at the 6-month (T1) and at the 12-month (T2) follow-up. Primary outcomes include whether or not they had been admitted to hospital and their length of stay. Secondary outcomes include the status of social functioning and problematic behavior (see Figure 2). The JOMP team psychiatrists provided participants diagnoses.

Variables concerning patient’s diagnosis were composed of ‘organic mental disorders (ICD-10, F0)’, schizophrenia, delusional disorders (ICD-10, F2)’, ‘mood (affective) disorders (ICD-10, F3)’, and ‘others’.

The Global Assessment of Functioning (GAF) [18] measured social functioning. GAF was developed for the overall assessment of psychological, social, and occupational functioning on a hypothetical continuum of mental health/illness rating 1 (persistently and serious impaired) to 100 (no symptoms, superior functioning). GAF reflects a need for multidimensional information and is known worldwide, has been translated into many languages and used in many outcome studies.

Social Behavior Schedule (SBS) [19] measured problematic behaviors. The SBS was designed for use with long-stay populations within a hospital or the community. It covers 21 behavioral areas, which describe the major difficulties exhibited by patients with long-term impairments that usually result in a dependence on or admission to a hospital. The SBS is scored using a Likert scale from 0 (no problem) to 4 (serious problem). It includes items relating to positive psychotic symptoms as well as negative behavioral items. Extracted by exploratory factor analysis from the SBS were four subscales that are behavioral-based rather than symptom-based: social withdrawal, thought disturbance, anti-social behavior and depressed behavior [20] and then was replicated with consistent results [21]. A high score on the scale indicates increased behavioral difficulty.

Activity logs were recorded for each visit during the service period to comprehend minutes of provided service to assess care amounts and contents (see Table 2). They were composed of service time (minutes), care categories, and IDs. If numerous team members’ dealt with the case at the same time, they record all participating members’ ID. Researchers counted the total amount of care by participant’s ID. Care categories were classified by care contents of psychiatric home visiting [22,23]. A list of care categories and summary of provided service per month is shown in Table 2. Each team recorded all their data on a computer database.

**Table 2:** **Transition of provided services time (minutes) between T0 and T2 for each client per month (N = 162)**

	**Before (n = 108)**	**1-month (n = 161)**	**2-month (n = 150)**	**3-month (n = 145)**	**4-month (n = 139)**	**5-month (n = 134)**	**6-month (n = 127)**	**7-month (n = 115)**	**8-month (n = 99)**	**9-month (n = 84)**	**10-month (n = 74)**	**11-month (n = 58)**	**12-month (n = 50)**	**Total**
Case management without clients	55.8	214.0	140.9	133.2	167.7	143.8	147.3	132.2	113.9	116.9	134.5	114.0	89.0	1703.1
Case management with clients	19.4	174.2	94.6	81.9	79.1	71.1	71.5	61.5	76.3	57.2	42.4	44.4	45.7	919.1
Assistance with daily living task	0.1	34.9	42.7	43.9	41.6	49.9	41.6	53.6	39.5	39.2	33.0	38.6	35.3	493.9
Communications and coordination	1.9	45.6	31.5	28.9	40.2	36.2	37.4	31.4	25.2	31.3	28.7	26.0	29.7	393.9
Family support	5.2	60.3	54.3	68.1	66.0	52.9	58.3	40.3	42.3	36.2	33.9	40.4	46.2	604.5
Medical support for psychiatric symptoms	1.4	41.6	52.0	48.1	74.7	81.5	66.5	85.5	49.4	68.4	44.6	42.1	34.8	690.6
Support for physical health	0.0	7.6	8.4	7.5	7.7	7.8	6.3	10.2	9.0	10.4	9.7	5.3	3.4	93.3
Social life and financial support	0.0	7.8	5.7	4.4	9.2	9.9	12.8	10.7	11.3	17.7	19.9	16.6	14.2	140.2
Housing services	0.0	5.6	2.6	3.2	3.2	5.4	8.1	8.4	3.0	4.5	4.5	11.3	2.6	62.3
Vocational and educational support	0.0	0.9	2.3	2.6	1.5	2.7	2.5	1.4	2.2	3.5	2.0	1.8	2.2	25.6
Total	83.8	592.5	435.0	421.8	490.9	461.1	452.2	435.4	372.0	385.2	353.0	340.4	303.1	5126.4

### Data analysis

To describe participant characteristics, we analyzed the baseline data of JOMP participants using percentages. Average length of stay and total time of provided services were calculated based on the date of service start, hospital admission and discharge from JOMP service. In order to test for significant effects of the intervention over time, repeated measures analysis of variance was performed. Data were analyzed using STATA 12.1 for Windows.

## Results

### Profile of the participants of JOMP

As might be expected slightly over half (55%) of 162 participants were diagnosed with schizophrenia. It was a fairly young sample with only 23% aged 50 to 59. The sample was evenly divided between men and women. The majority were not married (90%), were unemployed (84%) and lived with their family (66.9%). (see Table 3).

**Table 3:** **Participant characteristics at baseline (N = 162)**

	**N**	**%**
**Gender**		
Male	81	50.0
**Age**		
20-29	10	6.2
30-39	30	18.5
40-49	34	21.0
50-59	37	22.8
60-69	24	14.8
70-79	16	9.9
80+	11	6.8
**Marital status**		
Married	17	11.3
**Living situation**		
Living alone	53	33.1
**Occupation**		
Full-time employee	2	1.3
Part-time employee	4	2.6
Unemployed	136	89.5
**Diagnosis**		
Organic, including symptomatic, mental disorders	4	3.6
Schizophrenia, schizotypal and delusional disorders	89	80.9
Mood (affective) disorders	7	6.4
**Hospitalization during 18-months before utilizing the JOMP service**	27	16.7
**Medication during 18-months before utilizing the JOMP service**	29	17.9

### Admission rate at the follow-up period

**Figure 3:**
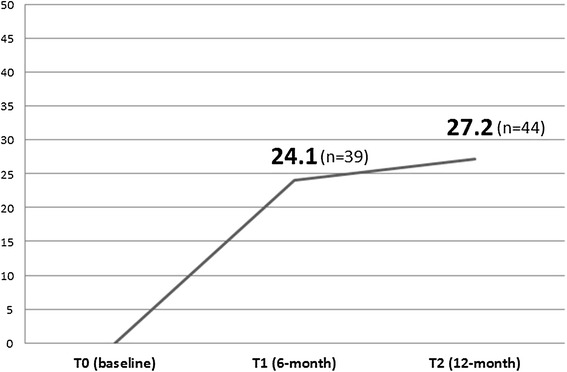
**Hospital admission rate at the follow-up period (N = 162).**

By the time of T1, 69 (42.6%) participants were discharged from JOMP, of which 16 (9.9%) were transferred into regular services, 39 (24.1%) readmitted to hospitals, and 14 (8.6%) for other reasons (dead or moved out of the catchment). By the time of T2, 108 (66.7%) participants had been discharged from JOMP; 42 (25.9%) were integrated into regular services, 44 (27.2%) had been readmitted to hospitals (including 3 admissions for physical problems), and 23 (14.2%) for other reasons. Thus the rates of hospital readmission were 24.1% at T1 and 27.2% at T2 follow up (see Figure 3). The average length of stay in the psychiatric hospital between T0 and T1 was 14.0 days (SD 41.4), T0 and T2 was 38.7 days (SD 84.7).

### Changes in social functioning and problematic behavior

The average baseline score at T0 for the GAF was 37.6 (SD 13.4) and the SBS score was 25.1 (SD 11.4). At T1 and T2 the GAF score improved and all SBS subscale scores were significantly reduced (see Table 4).

**Table 4:** **Changes in social functioning and problematic behavior at the follow-up period (N = 162)**

	**Baseline (T0)**			**6 months (T1)**			**12 months (T2)**			ANOVA*	
	**Mean**	**SD**	**n**	**Mean**	**SD**	**n**	**Mean**	**SD**	**n**	**F**	**p**
**Social functioning (GAF)**	37.6	13.4	144	42.5	15.7	146	41.9	16.2	109	20.0	<.01*
**Problematic behavior (SBS)**	25.1	11.4	152	21.6	12.2	152	18.5	12.8	57	17.5	<.01*
Social withdrawal	8.5	4.9		7.3	4.8		6.2	4.6		10.4	<.01*
Thought disturbance	7.4	5.0		6.6	4.8		5.8	4.6		9.3	<.01*
Depressed behaviour	2.6	2.6		2.2	2.3		1.7	2.0		5.9	<.01*
Anti-social behaviour	6.7	4.2		5.6	4.7		4.6	4.4		13.8	<.01*

SD, standard deviation. GAF, global assessment of functioning. SBS, social behaviour schedule.

*Repeated measure analysis of variance, significance at p < .05.

### Total time of provided services

Table 2 shows transition of provided services time (minutes) between T0 and T2 for each patient per month and total time. The JOMP staff provided long-term for case management (919.1 minutes per year per participant), prevention of exacerbations of psychiatric symptoms (690.6 min), support for his/her family (604.5 min), care for daily living (493.9 min) and maintaining interpersonal relationships (393.9 min).

## Discussion

The main goal of JOMP was to prevent readmission to the hospital. In this study the rate of participants’ readmissions within the first six months after joining JOMP was 24.1% and within twelve months it increased slightly to 27.2%. We compared the recent studies from Japan of patients who received regular outpatient care financed by public insurance. Uchiyama (2012) reported that of the 3,706 patients with schizophrenia from 525 hospitals in Japan 33.4% were readmitted within the first year after their discharge from a psychiatric ward [24]. Mayahara (2002) found that of the 30,071 patients with schizophrenia 30.7% were readmitted within one year after discharge from a psychiatric hospital [25]. Koyama (2004) reported that the 6-month readmission rate of 266 patients who were discharged from acute psychiatric wards was 24.1% [26]. While these outcomes look very similar to our study we must keep in mind that the participants of our study were consider high risk because of their lack of connecting to services. They had histories of past admissions and discontinued treatment after discharge. They most likely had a higher risk of readmission than study participants of Uchiyama, Mayahara and Koyama’s studies [24-26]. We have no studies about the readmission rates of high-risk patients in Japan. Our results of 24.1% at 6-months readmission rate in this high-risk population cautiously implies that JOMP contributed to a reduction in the readmission rate that was greater than those who received treatment through the regular Japanese outpatient clinic funded by public insurance.

In a previous study conducted in other countries about an assertive outreach program, Priebe et al. [12] reported 39% of the 487 participants were readmitted after nine months of AO program [12]. Firn et al. (2012) reported that 38% of 112 participants experiencing AO were readmitted to the hospital [24,27]. Brugha et al. (2012) reported 51% of the 1096 participants in the AO program were readmitted in the first year [10]. Carpenter Luce & Wooff (2011) reported the rate of inpatient readmissions during the 2 years before AO was 83.3% and after three years of the assertive outreach program it dropped to 56.5% [28]. Grinshpoon (2011) reported within 180 days of 908 psychiatric patient’s key discharge, 40% were readmitted who did not visit aftercare mental health clinic [29]. There are many differences in methodological approaches, treatment groups and country-specific differences and although we can’t directly compare these studies, the readmission rate of patients from JOMP appears to be lower.

The length of psychiatric hospital stay for the JOMP participants was 38.7 days (SD 84.7). The impact on length of hospital stay, of the Japanese ACT program, as reported in Sono’s research (2012) was 21.5 days (SD 52.8) [15] and Nishio [13] reported 56.7 days (SD 98.4) [13]. JOMP showed favorable outcomes in terms of hospitalization even if the level of social function among the participants at baseline (T0: *M =*37.6) was lower than the Japanese ACT studies and Uchiyama’s study. [13,15,24]. This also might be explained by the characteristics of the participants. JOMP participants had discontinued treatment for more than three months at the time of inclusion into our study: 72.8% of them had not been hospitalization during the previous 18 months and only 17.9% took some medications. Thus, the JOMP patients had severe disabilities, but they might have the potential to be stable and continuing living in the community once they could engage in outpatient care and continue to use the JOMP services.

The GAF baseline score (T0) of this study was 37.6 indicating impaired reality testing and communication and impairment in work, family judgment or mood and or major impairment in several areas, such as work or school, family relations, judgment, thinking, or mood. This score was lower than the findings in the Japanese ACT studies [13,15]. It suggests that the patients who have difficulties in maintaining contact with medical services could join the regular outpatient care if they have the support of the JOMP services.

Since participants of the ACT program had already connected with the program outpatient care and of JOMP did not receive regular medical treatment, this outcome seems to keep patients in the community similar to patients who were received regular outpatient care by public insurance.

The multi-professional team documented the amount of time they provided services. A high proportion of care time was devoted to case management and to conferences. Since the participants of this program had difficulties in using medical services, the JOMP project deliberately reached out to form relationships with participants through engaging them in medical care and social resources, managing and negotiating the various resources and tailoring optimal services for them and their families.

The goal of JOMP is to introduce patients to individualized services and to integrate them into the regular outpatient care system, but not to provide comprehensive team care over the long-term as did ACT and AO. Hence, one of the important functions of JOMP outreach team seemed to be outreaching to patients and introduction of service networks and this functioning seemed to work well to reduce the rate of hospitalizations and admission days.

Furthermore, being different from the usual care management, JOMP outreach teams also provided abundant direct care until the patients could independently engage in the usual outpatient care services. Among the direct care documented, the longest time was spent for ‘medical support for psychiatric symptoms’ and ‘family support’. Care for ‘assistance with daily living tasks’ and ‘communications and coordination’ were also delivered to the participants. The teams managed and prevented exacerbations of the participants’ symptoms, which was associated with a reduction of re-hospitalizations. Together with care for daily living and their communications, JOMP also contributed to the improvement of social functioning (GAF) and reduction of problematic behaviors (SBS). For the patients who have difficulties in keeping contact with services, it seems to be important to provide comprehensive direct care and care management to prevent patients from re-hospitalization and further impairment of their level of functioning.

Care for the family is important in the Japanese culture, as Sono [15] pointed out. In his study 76.8% of the participants were living with their families, and in our study there were 66.9% [15]. Compared with other studies in which the proportions of participants who lived with their families were 32.6-42.6% [7,10], the Japanese have a fairly high rate. In Japan, patients with mental disorders are generally cared for by parents or siblings. As the family members grow older, the capacity of the family to care for their disabled member becomes weakened. They are exhausted and overwhelmed. The JOMP outreach team establishes a relationship with the patient’s family and supports them through communication (active listening), providing information and caring for the patient. Considering the high proportion of care provided to family care, the family function seems to be an important factor in supporting the patients in community, particularly when one considers that in Japan, decision making resides as much if not more, within the family as it does with the individual.

There were several limitations to this research. First, we had no control groups for this study. Instead, we compared the outcomes with Japanese studies about patients who had received regular outpatient treatment by public insurance and Japanese ACT. But there were no comparable studies in Japan with patients who had difficulties in maintaining contact with medical services and had severe disabilities. Second, the distribution of psychiatric beds among the 47 prefectures was uneven with some areas of having more than others: with the western prefectures having more beds than the eastern and northern prefectures [30]. The locations of participating JOMP teams were distributed in northern, eastern, central and western part of Japan. However, there might be the influence of local differences on the results and samples, and this need to be examined in future research. It is not known if that influenced readmission or biased the sample. Since we collected data from 32 outreach teams in the 21 prefectures, it was difficult to set a comparable control group in this study. It is presumed that previous research would have comparable influences. Obtaining basic descriptive information of the programs and services offered by the 1,079 (65%) purely private psychiatric hospitals, [30], would enhance future research. Third, since this model project closed after 36 months, we could only engage the participants for 12 months. A longer observation period would, of course, provide a more realistic picture. Fourth, the instruments to measure patient’s symptom were limited and diagnoses had no established inter-rater reliability. In addition JOMP did not have outcome data about team characteristics in relationship to patient outcomes and team fidelity to the program goals. There are several studies showing that the characteristics of the team predicted participant’s outcome [6,10,12,28]. As of April 2014 JOMP was included in the National Health Insurance program in a limited way therefore an evaluation of JOMP team fidelity on readmissions must be examined.

## Conclusion

These results suggest that JOMP might be effective for keeping low readmission rates of patients who quit their psychiatric outpatient treatment, and for improving social function and delimiting problematic behavior. The JOMP teams provided long-term support for families. This should be one of the main functions of the care program. As of April 2014 JOMP was included in the National Health Insurance program in a limited way therefore an evaluation of JOMP team fidelity on readmissions must be examined.

## Consent

Written informed consent was obtained from the patient for the publication of this report and any accompanying images.
